# Embedding digital health education into pre-registration health degrees: a pilot evaluation of a train-the-trainer toolkit

**DOI:** 10.1186/s12909-026-10281-z

**Published:** 2026-09-03

**Authors:** Leanna Woods, Kerryn Butler-Henderson, Stephen Guinea, Melanie Haines, Samantha Robertson, Sophie Macklin, Melanie Kam, Clair Sullivan

**Affiliations:** 1 Queensland Digital Health Centre, Faculty of Health, Medicine and Behavioural Sciences, The University of Queensland, Level 5 Health Sciences Building, Royal Brisbane and Women’s Hospital Campus, Herston Queensland, Brisbane, 4006 Australia; 2 School of Nursing, Paramedicine, and Healthcare Sciences, Charles Sturt University, Wagga Wagga, Australia; 3 Digital Health Cooperative Research Centre, Sydney, Australia; 4 Faculty of Health Sciences, Australian Catholic University, Fitzroy, Australia

**Keywords:** Digital health, Health personnel, Education, Curriculum, Teaching, Students

## Abstract

**Background:**

A digitally-literate and future-ready healthcare workforce is essential to realise the benefits of digital health and address critical healthcare challenges. Embedding digital health content into health degree curricula is a strategic priority, yet integration is hindered by the absence of standardised, professionally endorsed curriculum and limited educator expertise. Following development of an educator toolkit, this pilot evaluation study aimed to explore: “How do educators and academic leaders perceive the appropriateness, usability, usefulness and feasibility of a toolkit designed to support integration of digital health content into pre-registration health curricula?”.

**Methods:**

A multi-method study was conducted across 17 Australian universities. Data collection conducted with educators involved structured online survey responses (*n* = 53) using a semi-structured conversation guide in a meeting and, after updating the toolkit with educator feedback, 60-minute online focus groups were conducted with academic leaders (*n* = 14). Survey responses underwent content and sentiment analysis with manual coding aligned with a pre-defined coding framework, while focus group data were analysed using content analysis.

**Results:**

Educators reported agreement that the toolkit was clear, well-structured, easy to navigate, and potentially effective in increasing their knowledge and confidence. Most statements rated positively for perceived appropriateness, usability, and usefulness. Statements with the highest agreement related to alignment between learning plans and outcomes (98%), educator knowledge and confidence (94%), and ease of navigation (92%). Lowest agreement related to planning assessments aligned with learning outcomes (60%), evaluating student capability (71%), and creating meaningful curriculum (70%). The toolkit was viewed as potentially applicable for multiple stakeholder groups, with greatest relevance for curriculum developers and educators. Academic leaders affirmed its appropriateness and timeliness, highlighting relevance and enthusiasm for future use. Ease of use was attributed to the clarity of topics aligned with learning outcomes and Bloom’s Revised Taxonomy. Five factors influenced feasibility: educator digital health literacy; curriculum and pedagogical guidance; assessment examples; toolkit structure and resources; engagement and evaluation.

**Conclusions:**

The toolkit could provide a practical mechanism for guiding educators and academic leaders in embedding digital health content into curricula within health education programs, supporting development of a future workforce equipped for evolving healthcare demands.

**Supplementary Information:**

The online version contains supplementary material available at https://doi.org/10.1186/s12909-026-10281-z.

## Background

Digital health has become pivotal in revolutionising and reshaping the way healthcare is delivered and is increasingly recognised as essential to address health system needs and workforce demands [1–4]. To realise the full benefits of digital health in improving health outcomes, a digitally literate, confident, and future-ready workforce is required [5]. Strengthening digital capabilities across the healthcare workforce has become a global priority, with a clear and growing need to equip the new, existing and emerging workforce cohorts [6].

Preparing the next generation of health professionals requires purposeful integration of digital health content into existing tertiary education health curricula [7]. Educators are likely to have limited expertise in this area [8]. Challenges facing educators include a lack of formal digital health training and experience, inconsistency across health disciplines, no nationally standardised or professionally endorsed set of learning outcomes or granularity to guide curriculum design and content integration, and crowded curricula [9, 10].

Several digital health capability frameworks have been developed to help guide workforce and education development, including the interprofessional Australian Digital Health Capability Framework (ADHCF) [11], the medical education DECODE framework [9] and the nursing TIGER framework [12]. While these frameworks outline the digital health capabilities required for contemporary practice, they provide limited practical support for curriculum implementation. In particular, existing frameworks provide limited direction on how capabilities can be translated into learning outcomes, curriculum content and assessment strategies. There is limited evidence on practical, educator-facing tools to help academics operationalise these frameworks across multiple pre-registration health disciplines.

To address these challenges, the Embedding Digital Health Education (EDHE) train-the-trainer toolkit (the toolkit) has been developed to support educators [13]. The toolkit is a publicly available resource hosted by the Australian Digital Health Agency and comprises 8 learning plans designed for educators of pre-registration health degrees. Each learning plan includes digital health topics mapped to learning outcomes, with practical guidance on curriculum integration, suggested teaching and learning activities and links to existing educational resources. The toolkit is intended to be flexibly adapted and embedded within existing curricula across health disciplines.

The toolkit was developed through national expert consensus, comprising an agreed set of digital health learning outcomes and topics to guide standardised digital health curriculum design for graduates of pre-registration health degrees (Table 1). Its purpose is to essentially “train the trainer”: to facilitate the introduction and integration of digital health content into pre-registration health degrees across a range of disciplines (e.g. nursing, medicine, pharmacy, allied health), build educator capability and ensure students of all health professions are exposed to foundational digital health knowledge and skills required to practice effectively in a technology-enabled healthcare environment. The toolkit comprises digital health topics aligned with both the Australian Digital Health Capability Framework [11] and Bloom’s Revised Taxonomy [14], further strengthened with Miller’s Pyramid [15] to create a consistent digital health curriculum that provides the foundations of digital health for a digitally-ready future healthcare workforce.

**Table 1:** Embedding Digital Health Education (EDHE) train-the-trainer toolkit for educators of health degrees in Australia: learning outcomes and associated digital health topics

Learning outcome	Topic	
1. Fundamental – Apply core technical concepts in digital health, including data, technologies, and information and knowledge management.	1.1 Role of digital health in improving quality, safety, access, and equity	
	1.2 Health data fundamentals	
	1.3 Electronic health records*	
	1.4 Telehealth*	
	1.5 Sensors and Internet of Things (IoT)	
	1.6 Standards and terminologies	
	1.7 Interoperability and integration	
	1.8 Health information exchange	
	1.9 Privacy and security	
2. Professional – Explain the importance of digital professionalism, including health literacy, legal and governance application, and ethical considerations, in the management and use of digital technologies and data.	2.1 Professional digital health literacy	
	2.2 Digital health literacy as a leader (workforce and consumer advocacy)	
	2.3 Consumer digital health literacy	
3. Influence – Analyse the social, economic, environmental, political, legal, regulatory, and ethical factors influencing digital health development and adoption.	3.1 Value-based digital health	
	3.2 Economics of digital health	
	3.3 Data and information governance*	
	3.4 Clinical governance	
4. Impact – Analyse the social, economic, environmental, political, legal, regulatory, and ethical factors in the co-design, development, management, use, delivery, and evaluation of digital health solutions.	4.1 Sociotechnical systems thinking	
	4.2 Co-design principles	
	4.3 Change management and leadership	
	4.4 Data and information governance*	
	4.5 Evaluation methods in digital health	
5. Transform – Critique digital health transformation initiatives and practices in Australia, and propose strategies to advance health equity, better consumer experience, greater health outcomes, workforce well-being, and high-value care.	5.1 Data informed decision-making	
	5.2 Data and information governance*	
	5.3 Data linkage	
	5.4 Digital health transformation in Australia	
6. Interface – Examine the role of the human–technology interface in clinical reasoning, decision-making, and care delivery, evaluating the risks and benefits to healthcare outcomes.	6.1 User-centred design	
	6.2 Clinical decision support	
7. Interprofessional – Critically reflect on the application of digital health technologies for interprofessional practice, including the scope of practice in your own discipline and in other disciplines.	7.1 Scope of own discipline	
	7.2 Scope of other disciplines	
8. Practice – Analyse the person-centred approach to the use of technologies in healthcare practice, emphasising respect, collaboration, and shared decision-making.	8.1 Information systems in healthcare	
	8.2 Electronic health records*	
	8.3 Telehealth*	
	8.4 Safe use of digital health	
	8.5 Health monitoring through technology	

* Topics appear under more than one learning outcome

We sought to answer the research question: How do educators and academic leaders perceive the appropriateness, usability, usefulness and feasibility of a toolkit designed to support integration of digital health content into pre-registration health curricula?

## Methods

### Design

A multi-method study design was implemented comprising surveys administered to educators of health degree programs, followed by focus groups conducted with academic leaders. The research team of eight (seven females, 1 male) included six PhD qualified researchers (LW, KBH, SG, MH, SR, CS) with extensive experience in qualitative and/or quantitative health service and digital health research, four registered healthcare professionals (LW, SG, SR, CS), a research assistant (SM) and a project manager (MK) both with medical science qualifications. The research is reported based on the consolidated criteria for reporting qualitative research (COREQ) [16] (Supplemental Table 1).

### Participants

Participants in this study were academic leaders and educators.

Academic leaders were recruited via self-selection through email correspondence sent to members of the Australian Council of Senior Academic Leaders in Digital Health (the Council). The Council convenes a national group of digital health academics from 37 member universities, aiming to foster and harness digital health scholarship, education, research and translation. Potential participants were informed about the project objectives during Council meetings, which aligned with the Council’s action plan to advance digital health workforce development and education [17]. Eligible academic leaders were those who have a comprehensive understanding of the current digital health curriculum at their respective universities, employed at an Australian university (one member per university), and willing to recruit educator participants onto the study.

Educator participants were recruited through purposive and snowball sampling from academic leader participants. Eligible educators were those responsible for developing or teaching curricula in at least one pre-registration health discipline program and employed at an Australian university. Participant exclusion criteria included those who were unwilling or unable to contribute to data collection within the study timeframe. For educators, previous experience with teaching digital health content was not considered. However, academic leaders were likely to be more engaged with digital health than their academic colleagues due to their involvement in the Council. Prior to the commencement of the study, some researchers (KBH, SG, MH, CS) had an established professional relationship with many participants through their ongoing involvement with Council operations.

### Data collection

**Fig. 1:**
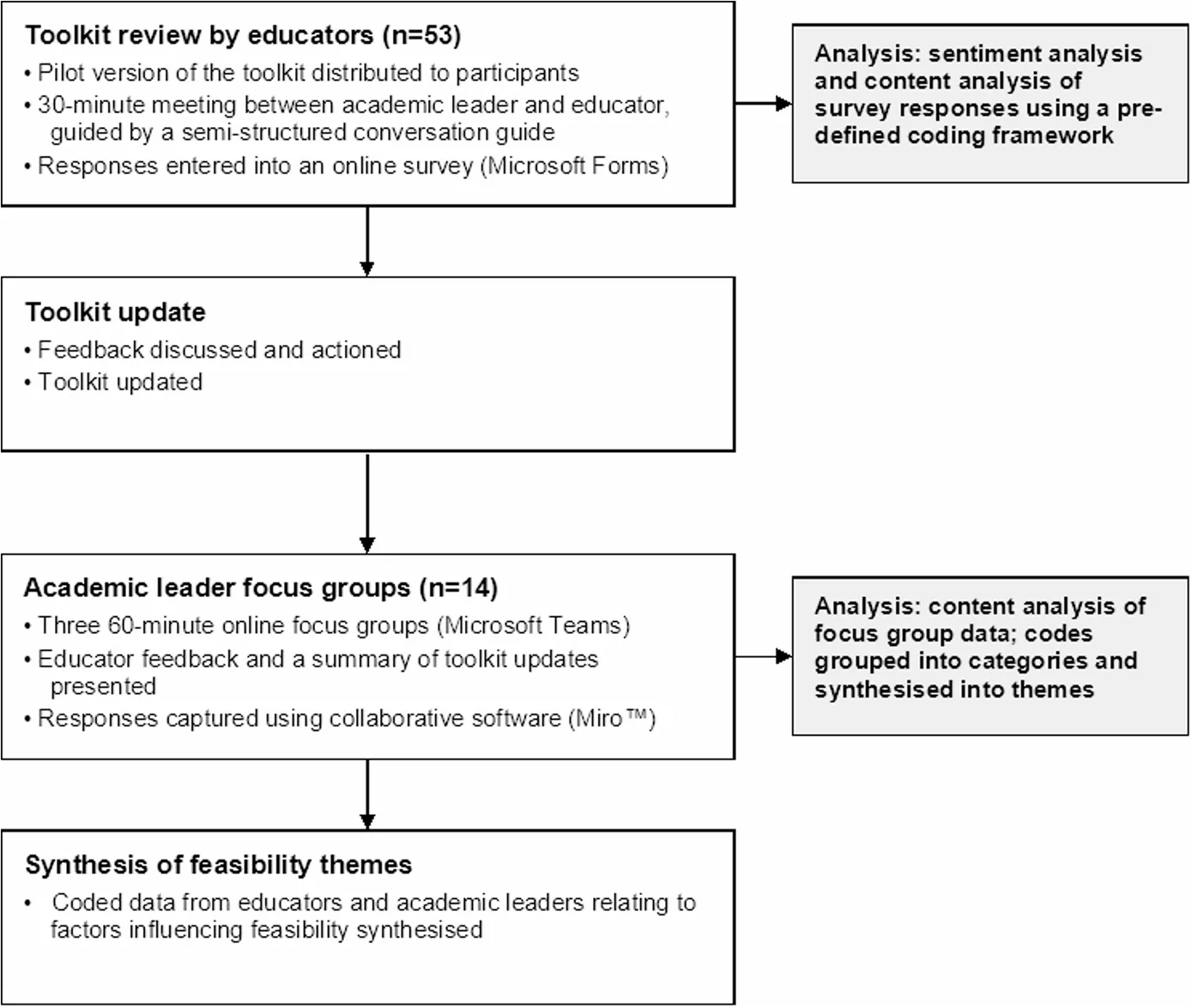
Data collection and analysis approach

Data collection occurred between July and September 2025 and involved surveys administered to educators, followed by focus groups conducted with academic leaders (Fig. 1). Prior to data collection, participants were emailed the same pilot version of the toolkit along with detailed instructions on how to engage with and contribute to the evaluation. Academic leaders were offered the opportunity to attend a non-mandatory information session with the research team to answer questions and clarify the process.

First, educator surveys were administered using Microsoft Forms and submitted by academic leaders following a scheduled 30-minute meeting between the academic leader and the educator. Meetings were conducted either online via university videoconferencing software or in person. The meeting was guided by a semi-structured conversation guide (Supplemental Table 2) developed and tested by the research team to elicit feedback on the toolkit. Academic leaders posed the evaluation questions to educators during the meeting and entered responses into the survey tool either contemporaneously or following completion of the meeting. Meetings were not audio or video recorded. Field notes were not forwarded to the research team. Data saturation was not considered. Survey responses were subsequently exported into Microsoft Excel for data management. Data obtained from the survey were considered by the research team and the toolkit was updated (see data analysis). Educator participants did not review the responses to the survey, nor did they provide feedback on the collated survey findings.

Second, focus groups with academic leaders were conducted via 60-minute online sessions via Microsoft Teams. To accommodate scheduling constraints, three alternative dates and times were offered, and participants self-selected into one session. Each focus group was facilitated by two researchers (KBH, SG) and supported by one or two moderators (LW, SR) who managed queries, resolved technical issues and recorded field notes. Participants were presented with the feedback from educators, a summary of the updates made to the toolkit and were asked two guiding questions: (1) What other information was shared in the meeting with educators that you couldn’t share in the survey? and (2) As a digital health expert, what were your thoughts on the toolkit, both positive and recommended changes? Responses were collected using Miro®^TM^ collaborative software with participants entering their input directly during the session. As the study focused on capturing key feedback rather than detailed discourse, focus groups were not audio or video recorded, and no transcripts were produced. Data were subsequently exported to Microsoft Excel for analysis.

### Data analysis

Survey responses from educators were analysed using two complementary approaches: sentiment analysis and content analysis. For sentiment analysis, each response to a statement was classified as positive, negative or neutral by two researchers (JH, LW). Positive or neutral classifications were interpreted as agreement with the statement, as neutral responses indicated no expressed disagreement or negative sentiment and therefore considered supportive or non-oppositional. Statements were organised into effectiveness domains of appropriateness, usability and usefulness.

Content analysis involved manual coding by researchers using a pre-defined coding framework (Table 2). Responses were double or triple coded when they addressed multiple categories. Initial coding was conducted in Microsoft Excel by two researchers (SM, JH), and subsequently validated by two additional researchers (LW, SR).

**Table 2:** Coding framework for survey responses provided by educator participants

Category	Code	Description
Learning outcomes	Clarity of learning outcomes	Learning outcomes clear/unclear. Topics fit/don’t fit learning outcomes. Intent of content is clear/unclear.
	Bloom’s level alignment	Level is/is not appropriate.
Learning plans	Level of content for educators	Level of content is/is not pitched at the correct level for educators – introductory e.g., too advanced/not advanced enough. Suitable/unsuitable resources.
	Level of content for students	The level is/is not pitched at the right level for students e.g., too advanced/not advanced enough. Suitable/unsuitable resources.
Toolkit	Ease of use	Difficult/easy to understand how to use toolkit, able/unable to use toolkit and apply to current curriculum.
	Relevance	Toolkit is/is not relevant to curriculum, students, educators, health discipline, education needs.
	Visual layout and structure of toolkit	Appropriate/inappropriate layout, appropriate/inappropriate images, logical/illogical flow, structural elements of the toolkit that require change.
	Improvements	Suggestions for improvement for toolkit.
	Implementation considerations	Factors to assist/enable or prevent/block successful implementation.

Working within each code, data were analysed by two researchers (LW, SR) and presented in a feedback table, which was presented to the research team in online workshops (KBH, SG, CS), discussed and actioned accordingly.

Focus group data from academic leaders were analysed by two researchers (LW, SR) after each focus group. Codes were grouped into categories, which were then synthesised into overarching themes to capture participant feedback with a count to reflect prominence of that theme. Data were summarised in a table, presented to the research team in an online workshop (KBH, SG, CS), discussed and classified as ‘noted’ or ‘actioned’ feedback. Following the completion of the focus groups, coded data from educators and academic leaders related to factors influencing feasibility were synthesised together.

## Results

### Participant demographics

In total, 17 universities which are members of the Council (*n* = 37) participated in the pilot evaluation, including educators (*n* = 53) and academic leaders (*n* = 14). Reasons for non-participation were not collected, and no participants withdrew from the study. Educators commonly held Associate Professor positions (*n* = 19), in the professional discipline of nursing (*n* = 10) with 15 or more years of higher education experience (*n* = 32) and had not previously taught digital health content (*n* = 32) (Table 3).

**Table 3:** Educator participant details (*n* = 53)

Category	Subcategory	Number (%)
Academic position	Casual academic	1 (2%)
	Lecturer	6 (11%)
	Senior lecturer	18 (34%)
	Associate Professor	19 (36%)
	Professor	6 (11%)
	Other	2 (4%)
	No response	1 (2%)
Professional discipline (multiple responses allowed)	Medicine	8 (15%)
	Nursing	10 (19%)
	Midwifery	2 (4%)
	Allied Health (not specified)	8 (15%)
	Pharmacy	3 (6%)
	Paramedicine	1 (2%)
	Dentistry	2 (4%)
	Nutrition and Dietetics	2 (4%)
	Speech Pathology	3 (6%)
	Physiotherapy	4 (8%)
	Occupational Therapy	3 (6%)
	Psychology	1 (2%)
	Exercise Physiology	2 (4%)
	Public Health	2 (4%)
	Digital Health	1 (2%)
	Health Information Management	1 (2%)
Number of years worked in higher education (*n* = 50)	1–5	2 (4%)
	6–10	7 (14%)
	11–14	9 (18%)
	15+	32 (64%)
Have you previously taught digital health or related content? (*n* = 50)	Yes	18 (36%)
	No	32 (64%)

### Perceived effectiveness of the toolkit among educators

Educators perceived the toolkit as broadly useful across multiple stakeholder groups, with the strongest potential relevance for curriculum developers and educators (Table 4).

**Table 4:** Educator perspectives on the perceived relevance of the toolkit to different stakeholder groups

Target audience	Frequency (*n* = 53, multiple responses allowed)
Curriculum leaders	41
Curriculum developers	46
Curriculum deliverers (educators)	45
Curriculum approvers (internal)	37
Accreditors (external)	34

Educators reported high levels of agreement that the toolkit was clear, well-structured, easy to navigate, and effective in increasing their knowledge and confidence, with most statements rated positively for appropriateness, usability, and usefulness (Table 5).

**Table 5:** Perceived effectiveness of the toolkit as reported by educators

Survey statement	Statement agreement (positive/neutral)	Sample participant quotes
Appropriateness of the toolkit		
The learning outcome is easy to interpret	83% (*n* = 43/52)	*Yes*,* they’re quite good. Bloom’s Taxonomy is clearly applied throughout*,* and you can see a progression emerging. The interpretation is straightforward*,* with very explicit use of cognitive verbs.* *Yes*,* the learning outcomes were very clear and easy to follow. I particularly valued the way [the figure in the toolkit document] presented them—it worked like a roadmap*,* visually mapping out the outcomes for each topic. This structure made the information more accessible and gave a strong sense of clarity*,* which I felt was really well put together.*
The verb of the learning outcome is at an appropriate level (using Bloom’s Revised Taxonomy)	90% (*n* = 47/52)	*I felt the verbs used in the learning outcomes were well chosen and aligned appropriately with Bloom’s Revised Taxonomy. It was clear that they reflected the right cognitive levels*,* and there was a noticeable progression in the level of challenge and expectations*,* which demonstrated a thoughtful alignment with Bloom’s framework.* *Yes*,* she thought they were good as they align well with the questions’ Bloom’s level and would be easy to integrate into their existing scaffolding.*
The content of the learning plans is pitched at the right level for my students	79% (*n* = 41/52)	*Mostly about right*,* but some beyond what might be expected for undergraduate health students.* *Yes*,* I can see that the content is pitched appropriately across the different stages of our four-year undergraduate degree. For example*,* outcomes that focus on understanding or describing a health system align well with the first or second year*,* where foundational knowledge is being developed. As students progress into the third and fourth years*,* the focus shifts toward applying knowledge and proposing solutions*,* which requires more advanced skills and higher-level thinking. This progression feels well-structured*,* and I can clearly see the applicability of the learning plan across all year levels.*
I can see opportunities to integrate digital health curriculum across the degree to achieve this learning outcome	79% (*n* = 41/52)	*Agree*,* very important to include*,* it should be basic knowledge for all health students and be prepared to work with digital.* *Yes*,* not all of these may apply*,* but I could incorporate three or four as introductory aspects of digital health*,* with the possibility of applying more later.*
I feel confident integrating the content of the digital health learning plans at an introductory level	75% (*n* = 38/51)	*Yes*,* absolutely. I feel confident integrating the content of the learning plans at an introductory level and look forward to using this resource once it becomes available.* *Disagree - need support to identify the how to do it*,* and the parts relevant to my students.*
Usability of the toolkit		
The learning plans are well structured and easy to follow	80% (*n* = 41/51)	*Yes*,* I found the learning plans clear and easy to follow. I particularly appreciated the consistency in structure across each topic*,* which made the material feel well-organized and predictable in a positive way. This consistency supported understanding and provided a smooth learning experience.* *Yes - had decent resources from reputable sources.*
The toolkit is easy to navigate	92% (*n* = 47/51)	*Yes*,* it’s clear. The use of colours*,* arrangement*,* and topic areas all help. It’s not too bulky and can be read easily.* *Yes*,* colour coding is good*,* and well signposted. Hyperlinks from table of contents appreciated.*
The learning plans and resources are logically linked to the learning outcomes	98% (*n* = 52/53)	*Yes - can be nuanced for individual disciplines.* *Each topic is clearly tied to a learning outcome and mapped to a capability domain. The progression also makes sense starting with the basic technical concepts and moving toward more advanced practice. That alignment makes the learning pathway feel coherent and intentional.*
The toolkit includes the necessary definitions and explanations are provided	84% (*n* = 42/50)	*Yes - good as a start.* *Yes*,* but he would like to see more definitions around the more advanced level topics. For example*,* he was unaware of socio-technical systems theory and would have liked more information.*
The toolkit is not overly time-consuming for participants	77% (*n* = 41/53)	*Not onerous - digestible in short form or long form.* *Depends - took some time to go through*,* but not surprised by this. Someone more advanced/more knowledge around digital health literacy would take less time.*
Usefulness of the toolkit		
The information within each learning plan makes it easy for me to create meaningful curriculum	70% (*n* = 37/53)	*Somewhat agree - more detail about how this can be used across the many disciplines in different settings. Relies heavily on user to contextualise it.* *Overall*,* yes*,* there are aspects of the plan that could align well with parts of the curriculum*,* particularly placement-based units*,* by showing how digital health impacts overall care. Students are likely to see the value of their learning once they experience placements*,* such as telehealth.*
The content and structure of the toolkit is likely to help the individual academics plan curriculum to meet the digital health learning outcomes	89% (*n* = 47/53)	*This is what [my university] and accreditation needs. Yes.* *Very useful scaffolding and structure for academics trying to plan curriculum and assessment to meet digital health learning outcomes.*
The toolkit increased my knowledge and confidence to deliver digital health content	94% (*n* = 47/50)	*The toolkit increased his appreciation of the value of digital health content. It helped him understand the key elements involved and prompted reflection on how this content could be implemented within the industry.* *I think it has strong potential to enhance both knowledge and confidence.*
The information within each learning plan allows me to build assessments to evaluate digital health capability in my student cohort	71% (*n* = 37/52)	*While good - providing more structured examples for someone who doesn’t work in digital space - also what to assess*,* what not.* *Not so much - not a lot of guidance on assessment. But good educators should be able to make this bridge.*
The content and structure of the toolkit is likely to help individual academics plan assessments to meet the learning outcomes	60% (*n* = 32/53)	*Somewhat agree - missing the ‘how’ to do it.* *No*,* need examples.*
The toolkit provided sufficient background knowledge to support my understanding of the digital health content	84% (*n* = 43/51)	*Yes*,* absolutely. The toolkit provides sufficient background knowledge*,* supported by extensive resources for each topic. To further strengthen knowledge translation*,* it may be helpful to include case scenarios.* *Disagree - going back to someone who doesn’t have digital health knowledge*,* there needs to be more structure and guidance/additional resources.*
The toolkit helped me understand how to teach digital health	86% (*n* = 44/51)	*Agree - all there*,* everything you’ve ever wanted to know in one package.* *It provides information on areas of digital health that should be included in the curriculum (i.e.*,* the “what”)*,* however there is very little information about the “how” of teaching digital health.*

Denominators vary due to missing data

Percentages are based on available (non-missing) responses

Educators valued the toolkit as a potentially useful resource and provided constructive feedback to further enhance its clarity and applicability. Participants acknowledged the relevance of the learning outcomes and suggested minor refinements, rewording for improved clarity, clarifying technical terminology, and ensuring accurate topic labelling. Educators highlighted the importance of confirming the constructive alignment between learning outcomes, the expected level of cognitive challenge (Bloom’s Revised Taxonomy) [14] and topics, which they viewed as a strength of the toolkit. To maximise future effectiveness, participants recommended expanding guidance on how learning outcomes can be effectively embedded across health degree programs. While most topics were considered appropriate, some were identified as advanced for pre-registration health degrees, offering an opportunity to tailor or stagger content for different learner cohorts.

### Perceived effectiveness of the toolkit among academic leaders

Academic leaders acknowledged the appropriateness and timeliness of the toolkit, noting its relevance and expressing enthusiasm for future use. The toolkit was considered easy to use, particularly due to the clarity of topics, and their alignment with learning outcomes and Bloom’s Revised Taxonomy [14]. Suggestions to enhance usefulness included removing resources that require external registration and providing descriptions and source information in the resources section to enable transparency. Participants recommended identifying priority learning outcomes for integration into curricula. To improve usability, feedback highlighted the need for additional content to support navigation, clearer guidance on educator expectations, and minor refinements to ensure ease of use.

### Factors influencing feasibility of a toolkit to support integration of digital health content into health curricula as reported by academic leaders and educators

Educators and academic leaders indicated similar insights to the feasibility of the future embedding of digital health into health degrees using the toolkit, reported across five themes.

#### Educator digital health literacy

Majority of educators that participated in this study had not previously taught, nor were currently teaching, digital health content in their role. Responses from both educators and academic leaders suggested variability in confidence and baseline knowledge to successfully deliver digital health content to students. To increase feasibility of the toolkit, educators “would require additional upskilling” (P33) through training and support to seamlessly integrate content without previous digital health exposure or background. A digital health training package was requested: “For successful integration at even an introductory level, professional development and support would be essential” (P1). Additionally, supplementing practical guidance with “introductory theory or frameworks to enrich educator understanding and confidence” (P25).

#### Curriculum and pedagogical guidance

Educators and academic leaders commonly identified a need for further guidance on incorporating the toolkit into health curricula. Some educators reported lower levels of confidence and “some anxiety” (P1) in implementing the toolkit successfully, partially due to the “already crowded curriculum” (P8). Although most educators believed that digital health “should be basic knowledge for all health students” (P4), and the toolkit was pitched at the right level for students, some indicated a few topics “may not align with the intended undergraduate level” (P25). Additional guidance was requested on tailoring materials to specific disciplines and scaffolding learning across year levels. Other factors for consideration included constructive alignment between learning outcomes, lesson plans and assessments, and sequencing of topics for implementation. Practical examples of “how educators can teach these concepts” (P10) across curricula, and specific teaching and learning material were requested by participants as the digital health content is novel for many. To increase feasibility of the toolkit, recommendations on how to effectively communicate concepts with “progressive complexity” (P5) to learners was desired, including templates, pre-learning and in-person materials, case studies and full lesson plans.

#### Assessment examples

Educators and academic leaders emphasised the desire for practical, engaging and innovative examples of assessment to be included in the toolkit to effectively evaluate digital health education: “The toolkit does not provide ready-made assessment tasks or marking guides, so academics must still design these from scratch” (P25). To increase feasibility, there was acknowledgment that assessments will need to be accompanied with marking criteria, rubrics, constructive alignment with Bloom’s taxonomy levels and learning outcomes. Detailed instructions to help academics plan assessments to “evaluat[e] digital health capability” (P27) was also requested by educators.

#### Toolkit structure and resources

Some educators identified the toolkit as comprehensive and well-structured, while others deemed it “overwhelming” (P12) when approached as a whole. To increase feasibility, participants addressed improvements to the toolkit structure, including developing a “digital version” (P14) of the toolkit to help with usability, navigation and maintenance of contemporary, scholarly and rigorous resources and ensure ongoing relevance. Educators identified the need for further detail and direction regarding the resources, with additional guidance on how to use the resources to achieve each of the learning outcomes suggested to improve toolkit feasibility.

#### Engagement and evaluation

Supporting a coordinated effort to communicate to educators the benefits and importance of incorporating the toolkit was reported as necessary to increase engagement across universities and disciplines (P24). Working with accreditation bodies will be necessary prior to formalising digital health content within curriculum, noting that digital health content will need to be mandatory and align with professional “accreditation requirements” (P34) and “map how these learning outcomes fit onto their professional standards” (P38). Guidance from relevant influential bodies would be beneficial in ensuring toolkit feasibility and that its elements are integrated into curriculum consistently across various contexts. Digital health education “champions … who can help to guide what needs to happen in the teaching and learning environment” (P41) was suggested to increase engagement and provide support across universities. To further identify the impact of the toolkit and “evaluate its effectiveness” (P37), it was recommended that a post-implementation activity be conducted.

## Discussion

Health professional education programs globally are facing the challenge of evolving legacy curricula into forward-facing educational programs that equip healthcare workers for the advent of digital health and artificial intelligence (AI). There are many strategies for this transformation, but limited evidence-based guidance for the operationalisation of these strategies. One of the biggest barriers to incorporating digital health into pre-registration curricula, is that most educators have limited experience and education themselves in digital health technologies. Prior research has shown that health professional educators often have limited confidence, knowledge and experience in teaching digital health [18, 19], a finding reflected in our study, with over 60% of Australian senior university educators reporting they had never taught digital health. This gap prompted the development of the toolkit to “train the trainers” and provide confidence and content to educators seeking to update their curriculum for the pressing issues of rapid technology uptake in healthcare.

Proactive digital health education and training, particularly in pre-registration health degrees, is necessary to enable engagement and effective use of digital health technologies [20, 21]. The focus of digital transformation in healthcare tends to remain on technologies rather than on developing workforce capabilities and competence in their application and use [22]. This narrow focus limits the potential for digital health to drive transformative change across health organisations. Strengthening digital health proficiency and competencies among healthcare professionals has proven to increase acceptance and use of digital health technologies across the workforce [22], and contributes to the broader goal of digital health transformation advancing the quintuple aim of healthcare [23].

Integrating digital health content into health degrees is identified as increasingly important, yet it remains underrepresented in health degree curricula globally [24–26]. While medical programs have begun addressing this gap by developing strategies to embed digital content into academic coursework [27], progress is slow and significant limitations persist [28]. For medical students, digital health courses are mostly elective, focus on a single area of digital health, and lack robust evaluation [29]. A recent scoping review up to 2024 found no evidence of nursing programs offering standalone courses in nursing informatics or digital health and identified no interventional studies on AI education at the undergraduate and graduate levels [30]. In Australia, digital health education is mostly prevalent in postgraduate courses, with education focused on specialisation or post-registration upskilling [20]. Successful integration of digital health content relies upon academic leadership to enhance educator literacy and provide guidance in implementing curriculum and adequate assessments. Digital health assessment remains uncommon in health profession education [19], and assessment of digital health competencies has been identified as a key challenge for educators [31]. Our findings suggest that educators seek support with both digital health content itself and with strategies for embedding and teaching it effectively.

The toolkit has potential to support a transdisciplinary approach to standardise digital health education for pre-registration health degrees, ensuring consistency across medical, nursing and allied health professions. Starting this journey at the pre-registration level and addressing the limitation of variable educator digital health literacy and educator knowledge would be aligned with the digital transformation of health systems [19]. To achieve standardisation, accrediting bodies should incorporate digital health capabilities into accreditation standards [6], recognising accreditation requirements as a key driver of curriculum design, implementation and sustainability in digital health education [31]. Additionally, ongoing evaluation is needed to measure the impact on practice and patient reported outcomes to ensure care improvement.

### Strengths and limitations

This study has several strengths. Its national reach across universities and the involvement of both educators and academic leaders provided perspectives from teaching, and curriculum design and governance. Participants represented a range of interprofessional health disciplines supporting broad applicability of the toolkit across health degree programs. Data analysis involved multiple members of the research team validating the coding and themes to enhance rigour and reliability.

Several limitations should be noted. First, participants self-selected into the study and included those affiliated with the Council, which may have introduced selection bias and may have resulted in an overrepresentation of participants with an interest in digital health. However, efforts were made to ensure diversity and representation within the study sample by inclusion of participants from multiple health disciplines and universities across different Australian states and territories. Second, sentiment analysis was based on subjective coding of qualitative responses. As neutral and positive sentiments were both classified as agreement, the results may overestimate positive findings and should be interpreted accordingly. Educator responses were not audio-recorded, and responses were mediated through academic leaders as part of the study design, which may have influenced the representation of educator perspectives. Third, as a pilot study, the toolkit has not been implemented for a sufficient duration to evaluate effectiveness or implementation outcomes. The next phase of the research will focus on implementing the toolkit across Australian universities, with plans for further longer-term evaluation. Finally, certain elements of the toolkit may be influenced by the structure of Australian health degree curricula and institutional requirements and may be limited to the Australian context, or require adaptation for use in other countries.

## Conclusions

Embedding digital health into pre‑registration health degrees is essential for developing a future‑ready workforce. By adopting a “train‑the‑trainer” approach, the toolkit has the potential to addresses current gaps in academic capability and provides a practical means to build educator confidence and competence in digital health. The toolkit offers a structured, scalable solution for educators seeking guidance on both digital health content and effective teaching strategies. Moving forward, the implementation plan encompassing toolkit adoption, curriculum embedding, teaching capacity enhancement and continuous evaluation, sets a clear and actionable pathway for strengthening digital capability across health education programs and preparing graduates for the evolving demands of contemporary healthcare. Further research is required to evaluate implementation of the toolkit across programs, and assess its impact on educator capability, curriculum change and student digital health competence.

## Supplementary Information

Supplementary Material 1.

Supplementary Material 2.

## Data Availability

The datasets generated or analysed during this study are not publicly available due to ethical and privacy restrictions but are available from the corresponding author on reasonable request.

## Abbreviations

The Council: Australian Council of Senior Academic Leaders in Digital Health
EDHE: Embedding Digital Health Education (into Health Degrees)
AI: Artificial Intelligence
P: Participant
