# Supplementary material for: Embedding digital health education into pre-registration health degrees: a pilot evaluation of a train-the-trainer toolkit
Source: BMC Med Educ. 2026 Sep 3;26:1562. doi: 10.1186/s12909-026-10281-z (PMC13628953; doi:10.1186/s12909-026-10281-z)
Supplement: Supplementary file 2 [file 12909_2026_10281_MOESM2_ESM.docx]

Supplementary Information 2

**Supplemental Table 2.** Feedback conversation guide for academic leaders to elicit educator feedback on the toolkit

| **Demographics** | 1. What is your current academic position? (casual academic, lecturer, senior lecturer, associate professor, professor, other) |
| --- | --- |
|  | 2. What is your health discipline? (nursing, medicine, allied health, public health, other) |
|  | 3. How many years have you worked in Higher Education (<1, 1-5, 6-10, 11-14, 15+) |
|  | 4. Have you previously taught digital health or related content? (Y/N) |
| **Learning outcomes** | For each learning outcome, please comment on the following: |
|  | 5. The learning outcomes is easy to interpret |
|  | 6. The verb of the learning outcome is at an appropriate level (using Bloom’s Revised Taxonomy) |
|  | 7. I can see opportunities to integrate digital health curriculum across the degree to achieve this learning outcome |
| **Learning plans** | Discuss your response to the following statements, including why you either agree or disagree. |
|  | 8. The learning plans are well structured and easy to follow |
|  | 9. The content level of the learning plans is pitched at the right level for my students |
|  | 10. The learning plans and resources are logically linked to the learning outcomes |
|  | 11. The information within each learning plan makes it easy for me to create meaningful curriculum |
|  | 12. The information within each learning plan allows me to build assessments to evaluate digital health capability in my student cohort |
|  | 13. I feel confident integrating the content of the digital health learning plans at an introductory level |
| **Toolkit** | 14. The content and structure of the kit is likely to help individual academics plan curriculum to meet the digital health learning outcomes |
|  | 15. The content and structure of the kit is likely to help individual academics plan assessments to meet the learning outcomes |
|  | 16. The kit provided sufficient background knowledge to support my understanding of the digital health content |
|  | 17. The kit increased my knowledge and confidence to deliver digital health content |
|  | 18. The kit helped me understand how to teach digital health |
| **Usability** | 19. The kit is easy to navigate |
|  | 20. The kit includes the necessary definitions and explanations are provided |
|  | 21. The kit is not overly time-consuming for participants |
| **Relevance to different types of stakeholders** | 22. The kit would be helpful for:   a. Curriculum leaders b. Curriculum developers c. Curriculum deliverers (educators) d. Curriculum approvers (internal) e. Accreditors (external) |
| **Improvements** | 23. Please provide your insights in relation to how the kit could be improved, e.g.:   a. What is missing b. Additional resources c. Structure, layout |
